# Supplementary figures and images for: The influence of roads on the fine-scale population genetic structure of the dengue vector Aedes aegypti (Linnaeus)
Source: PLoS Negl Trop Dis. 2021 Feb 26;15(2):e0009139. doi: 10.1371/journal.pntd.0009139 (PMC7946359; doi:10.1371/journal.pntd.0009139)

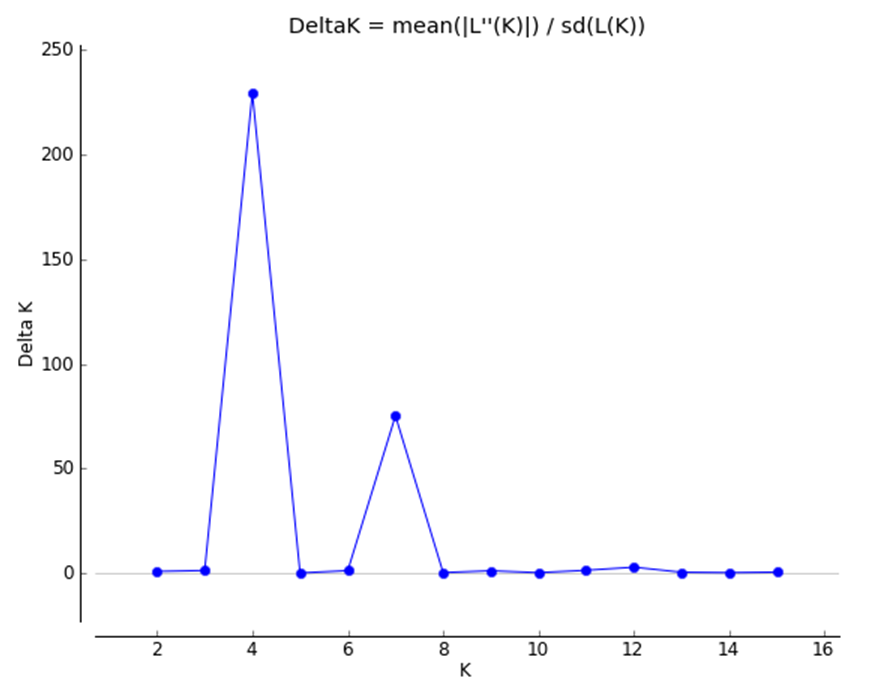

Supplement: S1 Fig — (TIF) [file pntd.0009139.s001.tif]

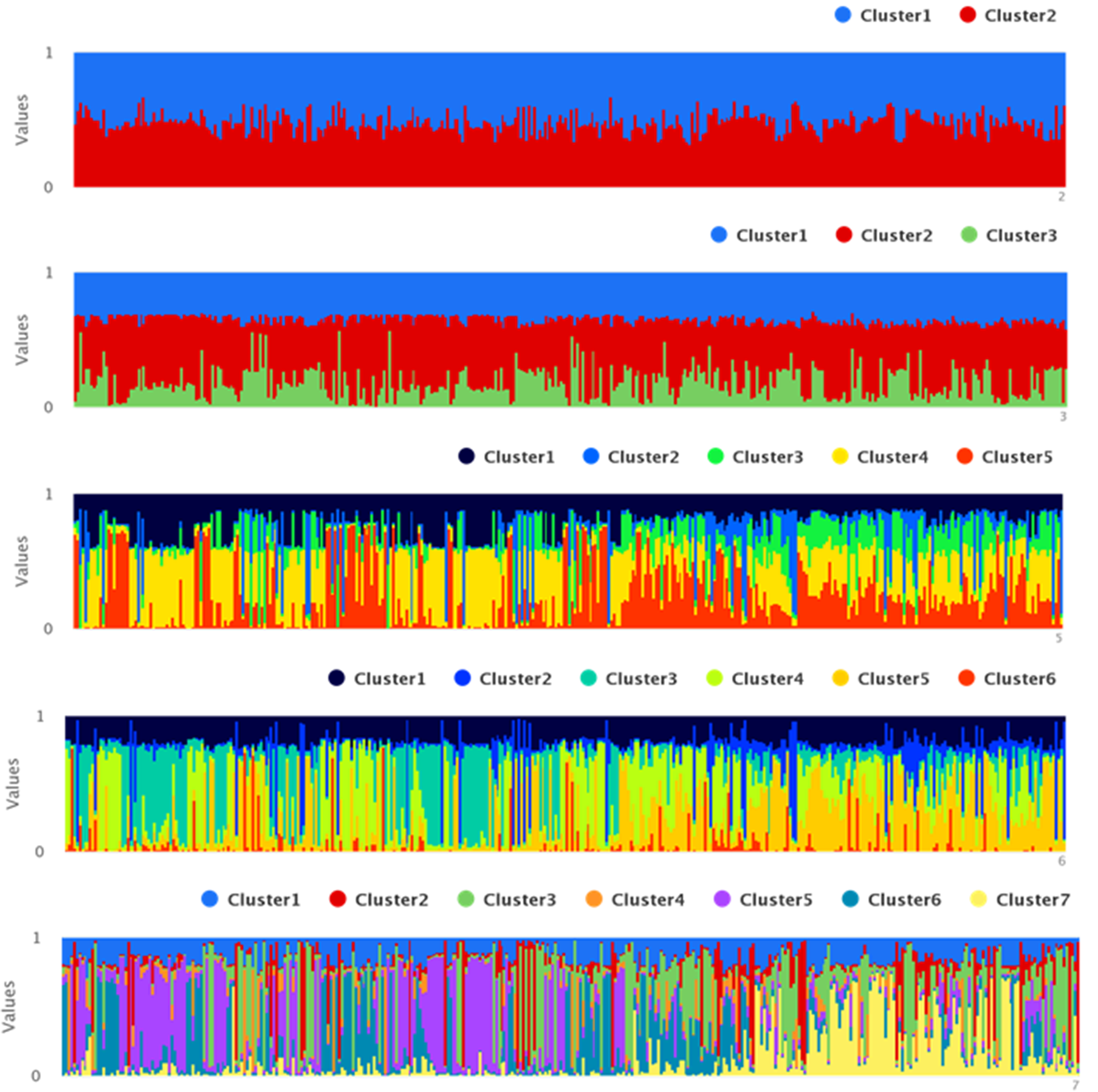

Supplement: S2 Fig — (TIF) [file pntd.0009139.s002.tif]

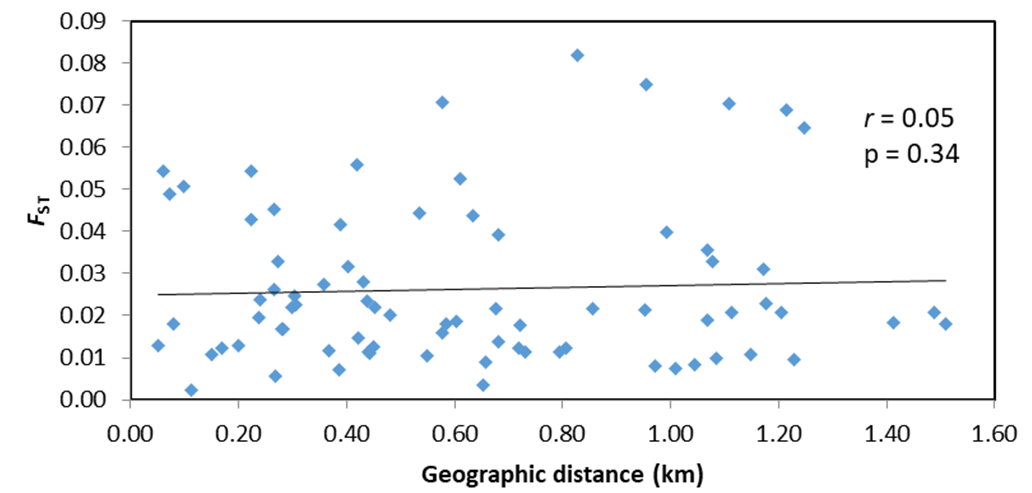

Supplement: S3 Fig — (TIF) [file pntd.0009139.s003.tif]

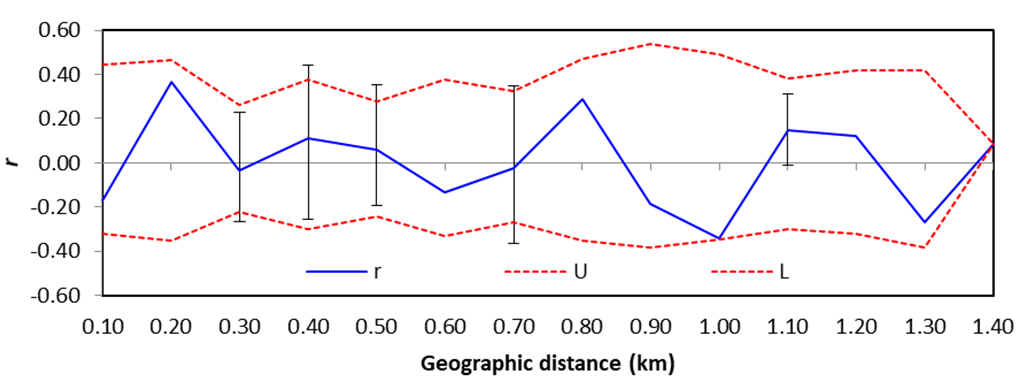

Supplement: S4 Fig — (TIF) [file pntd.0009139.s004.tif]
