# Supplementary material for: The influence of roads on the fine-scale population genetic structure of the dengue vector Aedes aegypti (Linnaeus)
Source: PLoS Negl Trop Dis. 2021 Feb 26;15(2):e0009139. doi: 10.1371/journal.pntd.0009139 (PMC7946359; doi:10.1371/journal.pntd.0009139)
Supplement: Attachment — Submitted filename: review_.docx [file pntd.0009139.s009.docx]

**PARV-D-20-00055**

review of Regilme et al 2020:

The influence of roads on the fine-scale population genetic structure of the dengue vector Aedes aegypti (Linnaeus)

**General comments:**

In this paper, the authors evaluate the influence of a primary road on the population’s genetic structure of *Aedes aegypti* mosquitoes. After mosquito collections, they use 11 microsatellite loci to genotype the mosquitoes. The results showed the presence of 4 genetic groups with a differentiation between north and south sites. Bayesian cluster analysis also showed the presence of 4 genetic clusters and autocorrelation analysis suggest a dispersal potential of the adult mosquitoes within a 200m spatial scale but a limited gene flow across the road.

This study was carefully planned and conducted and the results are clearly presented in 3 figures. I am totally in accordance with the discussion and conclusion and support the fact that the proposed strategy proposed by the authors could be an additional tool for vector control during dengue epidemics. I recommend this paper for publication in PNTD.

**Minor comments:**

**Abstract:**

L27: Larvae

**Methods:**

**Study site:**

It would be interesting to describe more the activity along EB and the traffic density along the day. Also are there any shaded areas or tree on EB. GPS location should be indicated.
