# Supplementary material for: The influence of roads on the fine-scale population genetic structure of the dengue vector Aedes aegypti (Linnaeus)
Source: PLoS Negl Trop Dis. 2021 Feb 26;15(2):e0009139. doi: 10.1371/journal.pntd.0009139 (PMC7946359; doi:10.1371/journal.pntd.0009139)
Supplement: Attachment — Submitted filename: Watanabe referaggio 2020.docx [file pntd.0009139.s010.docx]

**General comments**

The article "The influence of roads on the fine-scale population genetic structure of the dengue

vector Aedes aegypti" investigates population differentiation at small urban scale among the disease vector Ae. aegypti. The study design is interesting and the analysis of 11 microsatellites for mosquitoes sampled in 21 sites within a stretch of 2 km in the city of Manila allowed to show that the presence of major roads can work as barriers to gene flow causing small but consistent population genetic differentiation at local scale. Such information can be fundamental when programming vector control and defining mosquito population control zones.

**Introduction:**

Main comments:

I think the introduction section is well written and gives a good background to the study, although sometimes it appears repetitive.

Minor comments:

line 54: focusing on

line 86: are more likely possible

line 107-111: I would move this to M&M section

**Materials & Methods:**

Overall methods are well described although sometimes a bit confusing especially when describing mosquito sampling.

Minor comments:

Cite figure 1 when describing sampling sites.

Line 130 ongoing: I find this paragraph a bit confusing; could you please explain more clearly how you define the sample unit and the single sample site? How many sampling sites are there and how many household in each site? Where collections performed simultaneously in different sites or could some difference you observe also be due to temporal variation?

**Results:**

Minor comments:

When referring to sites you use a different numbering in Figure 1b compared to most part of the text and other figures where you use distinct numbering for northern (N1…) and southern (S1…) sites. Could you please use the same numbering overall (I would suggest to use the S1…N1…numbering which helps the reader to understand results immediately.

Line 219-221: was this significant?

Line 264: Maybe it could be interesting to have a look also on structure obtained with non optimal k-values; these could be represented in SM (but obviously add this information only if you think it is interesting?)

Line 272: Did you try IBD analysis also by separating northern and southern populations? Could results vary in these cases since you suggest an important impact of EB on differentiation?

**Discussion:**

Overall I think Discussion section is well written and deals with all results and explains their potential impact on control measures.
