# Supplementary material for: The influence of roads on the fine-scale population genetic structure of the dengue vector Aedes aegypti (Linnaeus)
Source: PLoS Negl Trop Dis. 2021 Feb 26;15(2):e0009139. doi: 10.1371/journal.pntd.0009139 (PMC7946359; doi:10.1371/journal.pntd.0009139)
Supplement: Attachment — Submitted filename: v2_FINAL_MS_Nica_PopGen_Response Letter_12182020.docx [file pntd.0009139.s011.docx]

18 December 2020

**Editor-in-Chief**

PLOS Neglected Tropical Diseases

Dear Editor-in-Chief,

We are pleased to submit the revised version of the submitted manuscript entitled “The influence of roads on the fine-scale population genetic structure of the dengue vector *Aedes aegypti* (Linnaeus)”. The manuscript was modified accordingly to the reviewers’ comments and suggestions. Moreover, the authors answered and explained carefully each of the feedback received. For your reference, the manuscript was revised accordingly with its corresponding line numbers.

**Reviewer #1** (Highlight in main manuscript - green)

|  | **Line no.** | **Comment** | **Response** |
| --- | --- | --- | --- |
| R1-01 |  | *Methods -Are the objectives of the study clearly articulated with a clear testable hypothesis stated?*  *-Is the study design appropriate to address the stated objectives?*  *-Is the population clearly described and appropriate for the hypothesis being tested?*  *-Is the sample size sufficient to ensure adequate power to address the hypothesis being tested?*  *-Were correct statistical analysis used to support conclusions?*  *-Are there concerns about ethical or regulatory requirements being met?*  yes to all no ethical concern | The authors are grateful for this feedback. |
| R1-02 |  | *Results -Does the analysis presented match the analysis plan?*  *-Are the results clearly and completely presented?*  *-Are the figures (Tables, Images) of sufficient quality for clarity?*  yes to all | Thank you for the comment. |
| R1-03 |  | *Conclusions -Are the conclusions supported by the data presented?*  *-Are the limitations of analysis clearly described?*  *-Do the authors discuss how these data can be helpful to advance our understanding of the topic under study?*  *-Is public health relevance addressed?*  yes to all | The authors are thankful for these feedback |
| R1-04 |  | *Editorial and Data Presentation Modifications?*  *Use this section for editorial suggestions as well as relatively minor modifications of existing data that would enhance clarity. If the only modifications needed are minor and/or editorial, you may wish to recommend “Minor Revision” or “Accept”.*  see attached file | Thank you for the valuable comments and suggestions in the attached file. |
| R1-05 |  | *Summary and General Comments*  *Use this section to provide overall comments, discuss strengths/weaknesses of the study, novelty, significance, general execution and scholarship. You may also include additional comments for the author, including concerns about dual publication, research ethics, or publication ethics. If requesting major revision, please articulate the new experiments that are needed.*  see attached file | Thank you for the comments in the attached file. |
| R1-06 |  | In this paper, the authors evaluate the influence of a primary road on the population’s genetic structure of *Aedes aegypti* mosquitoes. After mosquito collections, they use 11 microsatellite loci to genotype the mosquitoes. The results showed the presence of 4 genetic groups with a differentiation between north and south sites. Bayesian cluster analysis also showed the presence of 4 genetic clusters and autocorrelation analysis suggest a dispersal potential of the adult mosquitoes within a 200m spatial scale but a limited gene flow across the road. | The authors appreciate the insightful feedback from Reviewer #1. |
| R1-07 |  | This study was carefully planned and conducted and the results are clearly presented in 3 figures. I am totally in accordance with the discussion and conclusion and support the fact that the proposed strategy proposed by the authors could be an additional tool for vector control during dengue epidemics. I recommend this paper for publication in PNTD. | The authors highly appreciate the valuable feedback from Reviewer #1 . |
| R1-08 | L27 | Larvae | The authors corrected the word, please see L28 "larvae". |
| R1-09 |  | Methods:  Study site: It would be interesting to describe more the activity along EB and the traffic density along the day. Also are there any shaded areas or tree on EB. GPS location should be indicated. | The authors expanded this information. Please refer to L117-120. "It is one of the busiest roads in the city of Manila with heavy traffic congestion and high human population density. Some shaded areas such as trees can be found across EB. The mean width of the road (EB) sampled is 24.27 meters and its length is 2, 000 meters with coordinates of 14° 37′ 3″ N, 121° 0′ 4″ E. " |

**Reviewer #3** (Highlight in main manuscript - yellow)

|  | **Line no.** | **Comment** | **Response** |
| --- | --- | --- | --- |
| R3-01 |  | *Methods -Are the objectives of the study clearly articulated with a clear testable hypothesis stated?*  *-Is the study design appropriate to address the stated objectives?</br>*  *-Is the population clearly described and appropriate for the hypothesis being tested?*  *-Is the sample size sufficient to ensure adequate power to address the hypothesis being tested?*  *-Were correct statistical analysis used to support conclusions?*  *-Are there concerns about ethical or regulatory requirements being met?*  Overall this study was well thought and done. However, estimating migration rates between North and South will help clarify some pending questions.  The sample size of North and South sites combined is good. However, the sample size for individual sites is small. Therefore, conclusions based on comparisons between sites might not be reliable. I would compare individual sites with at least 20 samples. Sites with small sample size should be combined or left out of the analyses. | Thank you for the valuable comments and suggestions. The authors estimated the migration rates between North and South to help clarify the pending questions and as a supporting evidence. Please refer to the supplementary table (S4 table) for the detailed summary of the first generation migrants between North and South and within North and South. Please refer to the following lines  L206-214:  "To estimate the migration rate between the North and South sites and within North and South, we used the GENECLASS v2.0 [40]. For each individual *Ae. aegypti* in a population, we determined the probability that it belongs to its home population, probability of being a migrant across the road and the probability of being a migrant within North or South. We used the Bayesian criterion of [41] to identify the first generation migrants and the likelihood computation of Lhome/Lmax with a Monte-Carlo resampling algorithm [42]. We used the following parameter sets: 10,000 simulations and a threshold probability value of 0.05. In order to determine if the proportion of the migration rate across the road is lower than that within North or South, we performed Z-score for 2 populations proportions."  Please also see our response to R3-02 for the result of migration rate analysis.  About the number of sample size, the authors are thankful for the insightful suggestion to increase the sample size per site. Thus, the authors combined nearby sites with lower than 20 individuals, please refer to the updated summary at S1 table. The new sample size per site (n=13) range from 20 to 46 *Ae. aegypti* individuals*.* The authors also reanalyzed the data analysis, all changes in the manuscript regarding the updated results and discussion due to the revised sampling sites are highlighted in yellow in the manuscript. This change of sampling sites did not affect to our conclusion. |
| R3-02 |  | *Results -Does the analysis presented match the analysis plan?*  *-Are the results clearly and completely presented?*  *-Are the figures (Tables, Images) of sufficient quality for clarity?*  The results from individual based analyses (Structure) do not explicitly corroborate the the results from population based analyses (Fst).  I would suggest one more individual based analyses since your conclusions are based on them. It would be interesting to estimate migration rates between your North and South sites that seems to be differentiated. You could use migrate-N, BayesAss, Geneclass, etc. Then you would have a sense of historical or first and second generation migrants between these sites. | Thank you very much for the suggestion. The authors performed an additional individual-based analysis to estimate the migration rate using GENECLASS software. Please see the previous response to R3-01 for more detailed information about the migration rate analysis.  Please see the following L275-278 for the results of the migration rate analysis:  “We identified 94 individuals out of 376 (25%) as potential first-generation (F0) migrants (S4 Table), of which 26 (6.91%) were migrants across the road and 68 (18.08%) were migrants within South or North. The migration rate across the road (6.91%) was significantly lower than that within North or South (18.08%) based on the z-score test for two populations proportions at p <0.05.” |
| R3-03 |  | *Conclusions -Are the conclusions supported by the data presented?*  *-Are the limitations of analysis clearly described?*  *-Do the authors discuss how these data can be helpful to advance our understanding of the topic under study?*  *-Is public health relevance addressed?*  Again the results from individual based analyses support the conclusion of limited gene flow across the highway. However, the authors did not estimate migration rates among their sites.  Figure 2 indicates strong admixture within the sites and does not support limited gene flow.  Overall microsatelite data produces higher Fst estimates when compared to bi-allelic data and the conclusions are based on the Fst estimates and dendrogram. However, you forgot to show the support for each not on figure 2A. | Thank you, the authors acknowledge the suggestion. As mentioned previously, we performed estimate of migration rate. Please see our responses to R3-01 and R3-02.  Regarding the Fig 2 (Structure analysis), the aim of this analysis is to see if we can still observe genetic admixture even in a very fine spatial scale area as observed in previous studies done in a fine-spatial scale. The purpose was not to estimate gene flow across the road. The authors specified the purpose of the structure analysis, please refer to the following L89-99 “Furthermore, genetic analysis may also reveal co-occurrences of multiple genetic clusters at fine-spatial scale and their genetic admixture in mosquito individuals. For example, previous studies of *Ae. aegypti* revealed sympatric numerous genetic clusters (K = 3 to K= 16) from 30 km up to 2, 000 km [10-11,13,19], which might be due to the divergence from a single ancestry resulting into multiple genetic clusters over time and the random distribution of *Ae. aegypti* populations from nearby cities, regions or country. A recent study in the region of Metropolitan Manila, Philippines [13], discovered the probable number of genetic clusters of K = 4 in a fine spatial scale in Metro Manila, Philippines and genetic admixture in *Ae. aegypti* individuals. In this study, we narrow down the spatial scale up to 2 km to test if we could still observe multiple genetic clusters. This information is important prior to analysis of population genetic structure and gene flow because it can give background information on how the variety of genes are co-existing in a limited spatial scale. “  Microsatellite data may produce higher *F*_ST_ estimate as compared to bi-allelic data. However, out study did not use absolute values of *F*_ST_ across the road to support our conclusion of the barrier effect. In order to support our hypothesis of the barrier effect of roads, we used the AMOVA to test the significance of *F*_ST_ estimate between North and South. Also, we performed Mann-Whitney test to determine if the mean values of the pairwise *F*_ST_ within groups (North and South) and between groups are significantly different from each other to support our hypothesis of the barrier effect of roads. We also used the pairwise *F*_ST_ among the 13 sites for cluster dendrogram analysis to show the clustering of the genetically similar *Ae. aegypti* populations from North or South. The authors added the node value for Fig 1a and L261-263" The red numbers indicate the approximately unbiased (AU) p-values [42-43] and the green numbers are the standard bootstrap value (BP) [44] using the r package pvclust [45]. " |
| R3-04 |  | *Editorial and Data Presentation Modifications?*  *Use this section for editorial suggestions as well as relatively minor modifications of existing data that would enhance clarity. If the only modifications needed are minor and/or editorial, you may wish to recommend “Minor Revision” or “Accept”.*  Figure 1A show the supporting values for each node. | Thank you for the suggestion, the authors showed the supporting values for each node in Figure 1. Please see the previous response R3-03. |
| R3-05 |  | *Summary and General Comments*  *Use this section to provide overall comments, discuss strengths/weaknesses of the study, novelty, significance, general execution and scholarship. You may also include additional comments for the author, including concerns about dual publication, research ethics, or publication ethics. If requesting major revision, please articulate the new experiments that are needed.*  Overall, you can combine sites with small sample size that are near each other. You should also estimate migration rates (historical or recent). | The authors acknowledge this suggestion, the comment has been addressed in the previous response, please refer to R3-01. The small population sites that are neighboring to each other were combined and the data was reanalyzed. This modification of populations did not affect our conclusion. All new changes are highlighted yellow in the manuscript.  For the migration rates, please see our responses to R3-01 and R3-02. |
| R3-06 |  | General comments The article "The influence of roads on the fine-scale population genetic structure of the dengue vector *Aedes aegypti*" investigates population differentiation at small urban scale among the disease vector Ae. aegypti. The study design is interesting and the analysis of 11 microsatellites for mosquitoes sampled in 21 sites within a stretch of 2 km in the city of Manila allowed to show that the presence of major roads can work as barriers to gene flow causing small but consistent population genetic differentiation at local scale. Such information can be fundamental when programming vector control and defining mosquito population control zones. | The authors are grateful for the feedback. |
| R3-07 |  | Main comments:  I think the introduction section is well written and gives a good background to the study, although sometimes it appears repetitive | Thank you for the comment, we rechecked the introduction for repetition. Please refer to the deleted repetitive statement L106-107 “Previous study of [18], indicated a barrier effect of a highway to *Ae. aegypti* dispersal using all larval samples from water holding containers. Highways have continuous traffic without the presence of secondary roads that can be utilized as pathway for mosquito movement. “ |
| R3-08 | L54 | focusing on | Please see the revision, L55 focusing on |
| R3-09 | L86 | are more likely possible | Please see the revised L87 "are more likely possible" |
| R3-10 | L107-111 | I would move this to M&M section | We moved this to the methods section (L137-141): "To provide more information about the effect of roads, we collected adult mosquitoes and representative larval samples from each water holding containers across a primary road with presence of several smaller roads that can potentially carry mosquito migrants across. The sampling method of collection of both adult *Ae. aegypti* and larvae though labor intensive is more informative as compared to only larval samples. " |
| R3-11 |  | Materials & Methods: Overall methods are well described although sometimes a bit confusing especially when describing mosquito sampling. | The authors revised the mosquito sampling in the methods section. Please see L141-148: "Collections of adult and larval mosquitoes in each household were performed simultaneously in the different sites during the rainy season and high dengue cases, from June to September 2017. Adult mosquitoes were collected by installing commercially available mosquito UV light trap (MosquitoTrap, Jocanima Corporation, Las Piñas, Philippines) for 48 hours either inside or outside of each household. The UV light trap produces heat and CO_2_ gas attracting mosquitoes to enter into the capture net via a strong current from the ventilator [26]. In each household, one mosquito trap was installed. We also surveyed potential water breeding sites on each of the household premises and collected larvae when present." |
| R3-12 |  | Cite figure 1 when describing sampling sites | Please refer to the revised L132 "larval survey (Fig 1). " |
| R3-13 | L130 - ongoing | I find this paragraph a bit confusing; could you please explain more clearly how you define the sample unit and the single sample site? How many sampling sites are there and how many household in each site? Where collections performed simultaneously in different sites or could some difference you observe also be due to temporal variation? | Thank you for the comment. Please see the revised L129-136 "Each sampling site (n=13) is comprised of 1 to 5 smallest administrative division within the city (barangay; n = 35) and is defined as the population of this study. Households (n = 7 to 25 per sampling site) were selected based on their voluntary informed consent for mosquito adult collection and larval survey (Fig 1). The sampling unit of our study was the household defined as one unit of accommodation [24]. The geographic coordinates of the sampled households were recorded. In order to obtain a single geographic coordinate for each sampling site, we calculated the geographic midpoint [25] of sampled households in their respective sites. " and L141-143 "Collections of adult and larval mosquitoes in each household were performed simultaneously in the different sites during the rainy season and high dengue cases, from June to September 2017. " |
| R3-14 |  | Results:  Minor comments: When referring to sites you use a different numbering in Figure 1b compared to most part of the text and other figures where you use distinct numbering for northern (N1…) and southern (S1…) sites. Could you please use the same numbering overall (I would suggest to use the S1…N1…numbering which helps the reader to understand results immediately | Thank you for the suggestion. Please see the revised Fig 1. The authors used the same numbering to refer to the results, North (N1, N2…) and South (S1, S2…). |
| R3-15 | L219-221 | was this significant? | Yes, please see the revised L234-236 "All 13 sites displayed significant non-conformance to Hardy-Weinberg equilibrium (He > Ho) after Bonferroni correction …" |
| R3-16 | L264 | Maybe it could be interesting to have a look also on structure obtained with non optimal k-values; these could be represented in SM (but obviously add this information only if you think it is interesting?) | Thank you for the suggestion, we performed barplot analysis also in the non-optimal k-values and we also observed genetic admixture. Please refer to S2 Fig (Supplementary file) for the barplot of the non-optimal k-values. |
| R3-17 | L272 | Did you try IBD analysis also by separating northern and southern populations? Could results vary in these cases since you suggest an important impact of EB on differentiation? | Yes, we also performed IBD analysis by separating North and southern populations and we found also non-significant results. The authors assume that the results will not vary. Please see the added L282-283 "Non-significant results were also obtained in the northern populations (r = 0.03, p-value = 0.39) and in the southern populations (r = 0.29, p-value = 0.11)." |
| R3-18 |  | Discussion: Overall I think Discussion section is well written and deals with all results and explains their potential impact on contro`l measures. | Thank you for the feedback. |

**Editor’s comments (Highlight in main manuscript - blue)**

|  | **Line no.** | **Comment** | **Response** |
| --- | --- | --- | --- |
| E1-01 |  | We note that the following authors have not yet confirmed their authorship on this manuscript: Maria Angenica Fulo Regilme, Divina M. Amalin.  At this point this request is not urgent; we have re-sent their co-author confirmation email containing an auto-confirmation link for them to click, and we will reach out to them at a later stage if we don't hear from them. We wanted to mention this in case you are aware of them experiencing technical issues with confirming authorship via the link. If so, please do ask them to contact us directly via email and we'd be happy to assist. | Thank you for informing us about this.  The following authors have problems receiving the confirmation email.  We request to resend a confirmation email in the following email addresses:  Maria Angenica Fulo Regilme  [mafregilme@gmail.com](mailto:mafregilme@gmail.com)  [nica.regilme@gmail.com](mailto:nica.regilme@gmail.com)  Divina M. Amalin  [dmamalin@gmail.com](mailto:dmamalin@gmail.com)  [divina.amalin@dlsu.edu.ph](mailto:divina.amalin@dlsu.edu.ph) |
| E1-02 |  | We do not publish any copyright or trademark symbols that usually accompany proprietary names, eg (R), (C), or TM (e.g. next to drug or reagent names). Therefore please remove all instances of trademark/copyright symbols throughout the text, including Qiagen® on page 7, RNALater™, NanoDrop™ and Takara Ex Taq™ on page 7. | The authors removed all copyright and or trademark symbols throughout the manuscript. Please refer to the highlighted changes in blue, L157-166 Qiagen, RNALater NanoDrop and Takara Ex Taq |
| E1-03 |  | Please remove the figures embedded in your manuscript file leaving only the separate figures files. If you are using a LaTeX source file, please use \includegraphics to remove the figures from your PDF. | All figures embedded in the revised and clean manuscript were removed. Updated Fig 1 was uploaded separately as figure file. |
| E1-04 |  | ##Copyright  Please note that all figures and supporting information files will be published under the Creative Commons Attribution License (creativecommons.org/licenses/by/4.0/). Under this license, authors retain ownership of the copyright for their article, but allow anyone to download, reuse, reprint, modify, distribute, and/or copy articles in PLOS journals for any reason, even commercially, so long as the original authors and source are cited. Permission is not required from PLOS or authors to reuse any content as long as proper attribution is provided.  We ask that you please confirm that you either created the following or have written permission from the copyright holder to publish them under a CC-BY license: Figure 1.  We have noticed that your manuscript contains a map as one of your figures. Figure 1. Please ensure that this map was published under a CC-BY license (http://journals.plos.org/ploscompbiol/s/content-license) - maps licensed under any other license will NOT be compatible with our full re-use CC-BY license.  The following websites are good examples of where you can source open access or public domain maps:  * World of Maps - Many, but not all, of the maps are in the public domain so please check the usage rights before downloading. (http://www.worldofmaps.net)  * U.S. Geological Survey (USGS) - All maps are in the public domain. (http://www.usgs.gov) (http://glovis.usgs.gov/),  (http://landsatlook.usgs.gov/)  * PlaniGlobe - All maps are published under a Creative Commons license so please cite “PlaniGlobe, http://www.planiglobe.com, CC BY 2.0” in the image credit after the caption. (http://www.planiglobe.com/?lang=enl)  * Natural Earth - All maps are public domain. (http://www.naturalearthdata.com/about/terms-of-use/)  * NASA - All maps are public domain. (https://eol.jsc.nasa.gov/),  (http://earthobservatory.nasa.gov/) (http://landsat.gsfc.nasa.gov/images/)  * Wikimedia Commons - Many, but not all, of the maps are licensed under compatible licenses so please check the usage rights before downloading. (https://commons.wikimedia.org/wiki/Atlas_of_the_world) | The authors acknowledge the copyright rules. Thus we revised the map used in Fig 1, please see the newly uploaded fig1. Please refer to the added caption L258-259 “The map was created using ArcGIS version 10.2.2 from <http://landsat.gsfc.nasa.gov/images/>. “  Landsat (<http://landsat.gsfc.nasa.gov/images/>) is listed as a public domain and open access so we used it redraw the map. The newly redrawn map on Fig 1 complies with the CC-BY license. |

**References**

Balingit JC, Carvajal TM, Saito-Obata M, Gamboa M, Nicolasora AD, Sy AK, Oshitani H, Watanabe K. Surveillance of dengue virus in individual *Aedes aegypti* mosquitoes collected concurrently with suspected human cases in Tarlac City, Philippines. Parasites & Vectors. 2020 Dec;13(1):1-3.

Piry S, Alapetite A, Cornuet JM, Paetkau D, Baudouin L, Estoup A. GENECLASS2: a software for genetic assignment and first-generation migrant detection. Journal of heredity. 2004 Nov 1;95(6):536-9.

Rannala B, Mountain JL. Detecting immigration by using multilocus genotypes. Proceedings of the National Academy of Sciences. 1997 Aug 19;94(17):9197-201.

Paetkau D, Slade R, Burden M, Estoup A. Genetic assignment methods for the direct, real‐time estimation of migration rate: a simulation‐based exploration of accuracy and power. Molecular ecology. 2004 Jan;13(1):55-65.

Shimodaira H. An approximately unbiased test of phylogenetic tree selection. Systematic biology. 2002 May 1;51(3):492-508.

Shimodaira H. Approximately unbiased tests of regions using multistep-multiscale bootstrap resampling. The Annals of Statistics. 2004;32(6):2616-41.

Felsenstein J. Confidence limits on phylogenies: an approach using the bootstrap. evolution. 1985 Jul;39(4):783-91.

Suzuki R, Shimodaira H. Pvclust: an R package for assessing the uncertainty in hierarchical clustering. Bioinformatics. 2006 Jun 15;22(12):1540-2.

The authors acknowledge the editors and the reviewers for their helpful comments and suggestions thus improving the overall manuscript. We are looking forward that these revisions will warrant the acceptance for publication in your prestigious journal, PLOS Neglected Tropical Diseases.

Sincerely Yours,

**Dr. Kozo Watanabe**, Professor

Center for Marine Environmental Studies (CMES)

Ehime University, Bunkyo-cho 3, Matsuyama, 790-8577, Japan

Email: watanabe.kozo.mj@ehime-u.ac.jp
